# Supplementary material for: Sex and Age Impact CD4+ T Cell Susceptibility to HIV In Vitro through Cell Activation Dynamics
Source: Cells. 2023 Nov 23;12(23):2689. doi: 10.3390/cells12232689 (PMC10706042; doi:10.3390/cells12232689)
Supplement: Supplementary file 1 [file cells-12-02689-s001.zip › cells-2688871-supplementary.pdf]

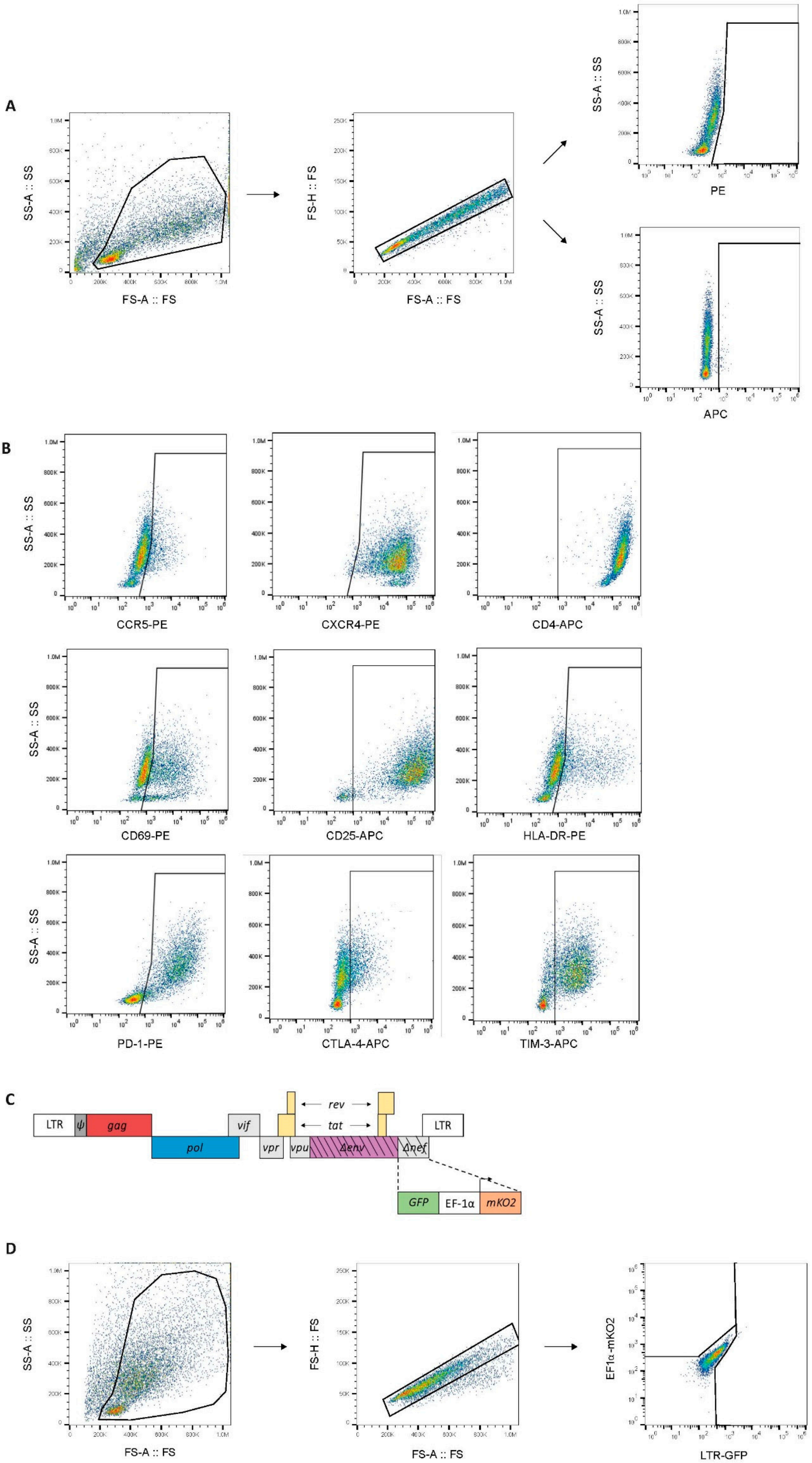
A
SS-A :: SS
FS-A :: FS
FS-H :: FS
FS-A :: FS
SS-A :: SS
PE
SS-A :: SS
APC
B
SS-A :: SS
CCR5-PE
CXCR4-PE
CD4-APC
SS-A :: SS
CD69-PE
CD25-APC
HLA-DR-PE
SS-A :: SS
PD-1-PE
CTLA-4-APC
TIM-3-APC
C
LTR
ψ
gag
pol
vif
vpr
rev
tat
vpu
Δenv
Δnef
LTR
GFP
EF-1α
mKO2
D
SS-A :: SS
FS-A :: FS
FS-H :: FS
FS-A :: FS
EF1α-mKO2
LTR-GFP

Figure S1. Flow cytometry gating strategies and HIV GKO genomic structure. (**A**) Gating strategy for cell surface marker assessment. Living cells were gated on SS-A vs FS-A plot and single cells were selected on FS-H vs FS-A plot. Marker expression was assessed based on fluorochrome expression, *i.e.* PE and APC. (**B**) Gating example of stained cells for each surface marker assessed. (**C**) HIV GKO is a dual reporter expressing LTR-controlled GFP and EF1-α-controlled (constitutive) mKO2 in *nef* ORF and harboring deletion in *env*. (**D**) Gating strategy for infection monitoring. Living cells were gated on SS-A vs FS-A plot and single cells were selected on FS-H vs FS-A plot. Susceptible cells were measured based on mKO2 expression, permissive cells based on GFP expression and latent cells based on mKO2 expression and absence of GFP.

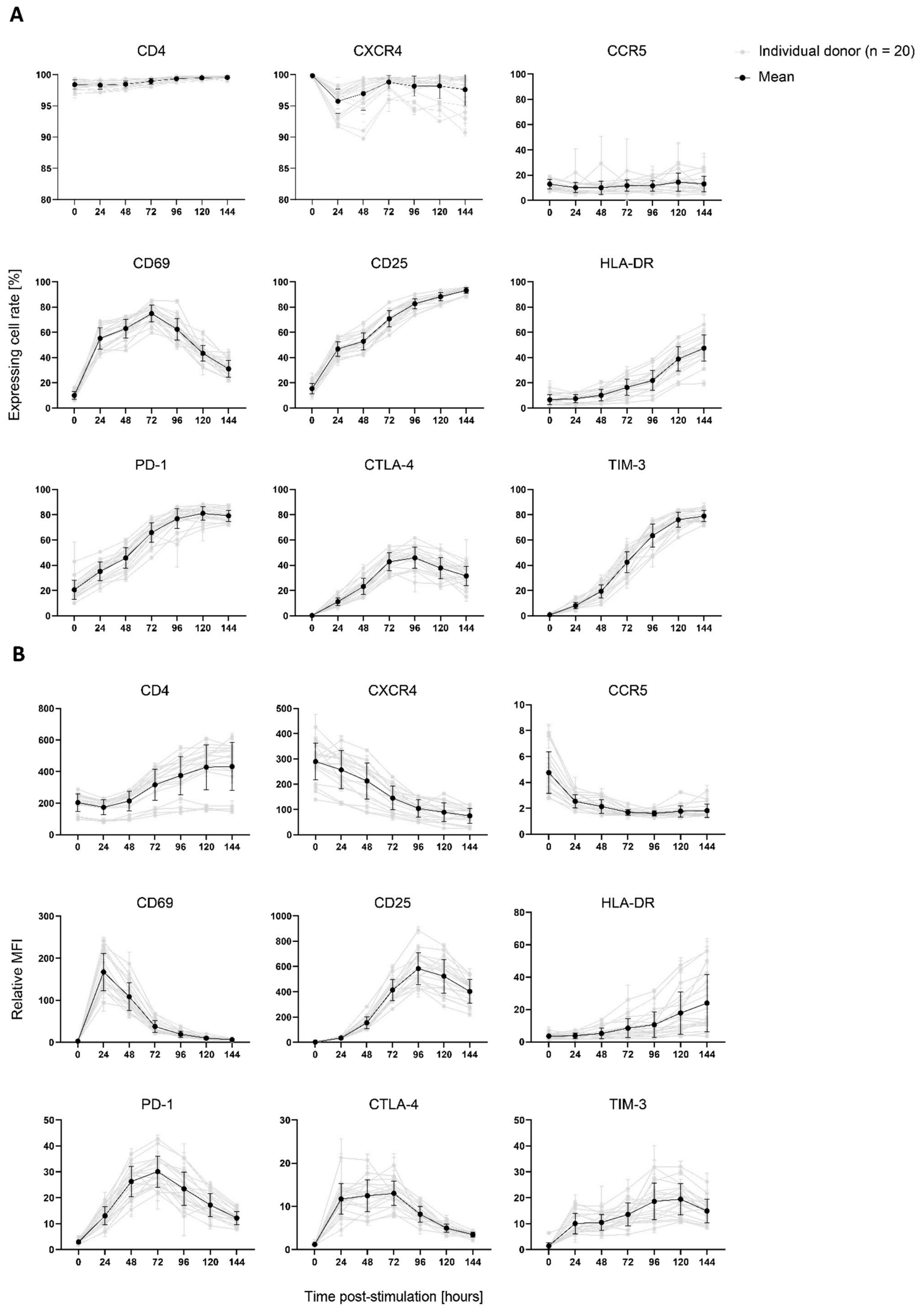

Figure S2. Surface marker expression over time post-stimulation for HIV entry and activation markers and immune checkpoint inhibitors. Grey lines represent expression kinetics obtained for each individual donor and is the mean of biological duplicates. Black line represents the mean of all donors. Error bars represent the SD. (**A**) Proportion of expressing surface marker (**B**) Global expression level was assessed as relative MFI, normalized by the corresponding non-stained control.

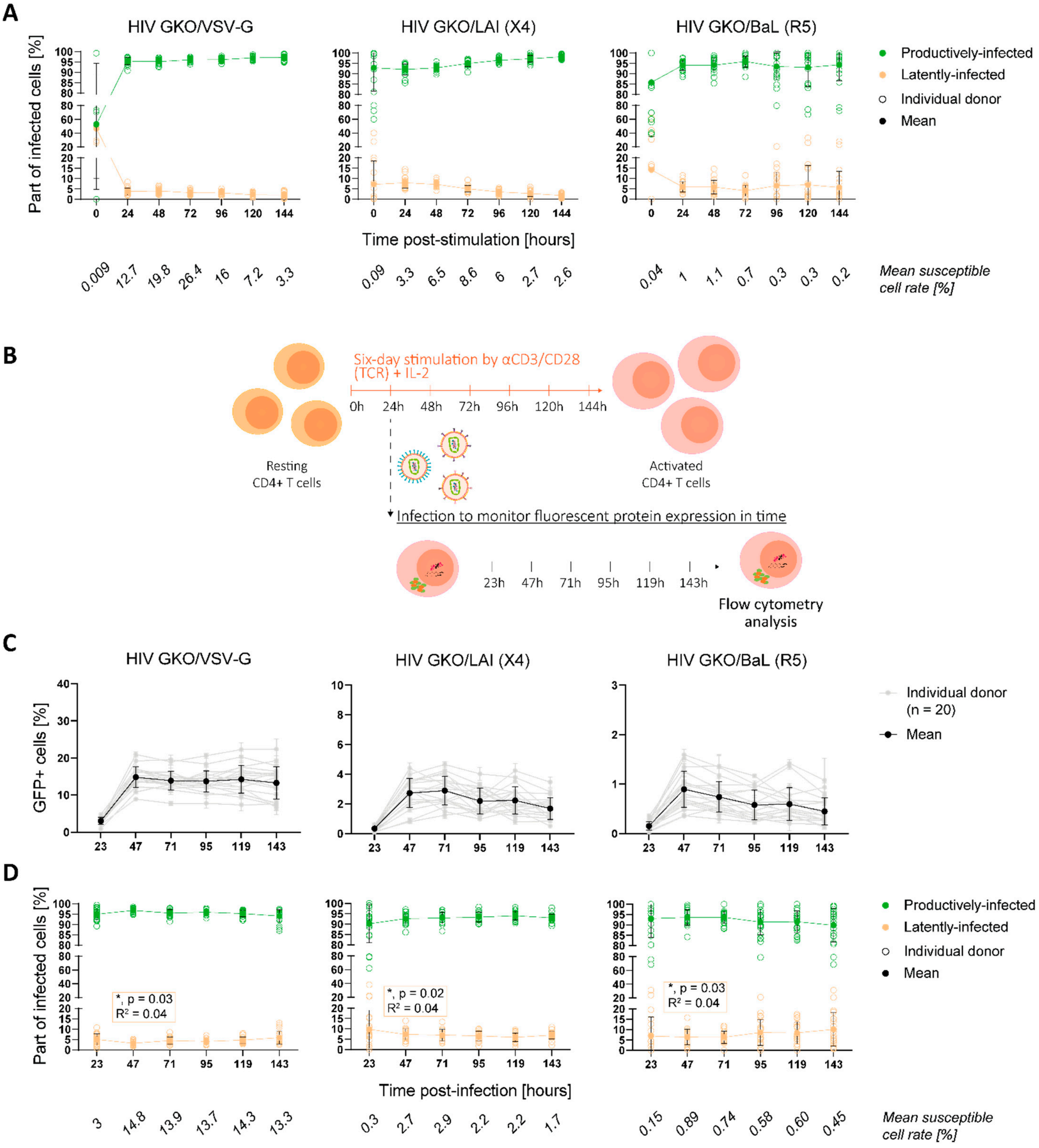

Figure S3. HIV latent infection. Proportion of infected cells upon infection with HIV GKO vectors harboring distinctive envelopes (VSV-G, LAI (X4), BaL (R5)), and divided as productive infection (mKO2+ GFP+ cells; green) and latent infection (mKO2+ GFP-; orange). Dots represent mean of biological duplicates per individual donor and lines represents the mean of all donors. (**A**) Proportion of latently and productively infected cells over time p.-s. Cells were stimulated for 6 days and cells were infected each 24h. Levels of infection success, as measured by mKO2+ cells 47h p.-i., are indicated at the bottom of the graphs. (**B**) Experimental workflow used to assess GFP expression stability over time in mKO2+ infected cells. Resting CD4+ T cells were stimulated for 24h with anti-CD3/CD28 in presence of IL-2 and infected with the three HIV GKO reporters (VSVS-G, LAI (X4), BaL (R5)). mKO2 and GFP expression were measured by flow cytometry at 23h, 47h, 71h, 95h, 119h and 144h p.-i. (**C**) Stability of viral protein expression over time was assessed by rate of GFP+ cells. Grey lines represent infection kinetics obtained for each individual donor and are the mean of biological duplicates. Black line represents the mean of all donors. Error bars represent the SD. (**D**) Proportions of latently (GFP-) and productively (GFP+) infected cells in mKO2+ cells over time p.-i. The proportion of mKO2+ infected cells (*i.e.* susceptible cells) is indicated at the bottom of the graphs. Linear regression analysis was performed on latently infected cells over time; results are indicated in the colored boxes.

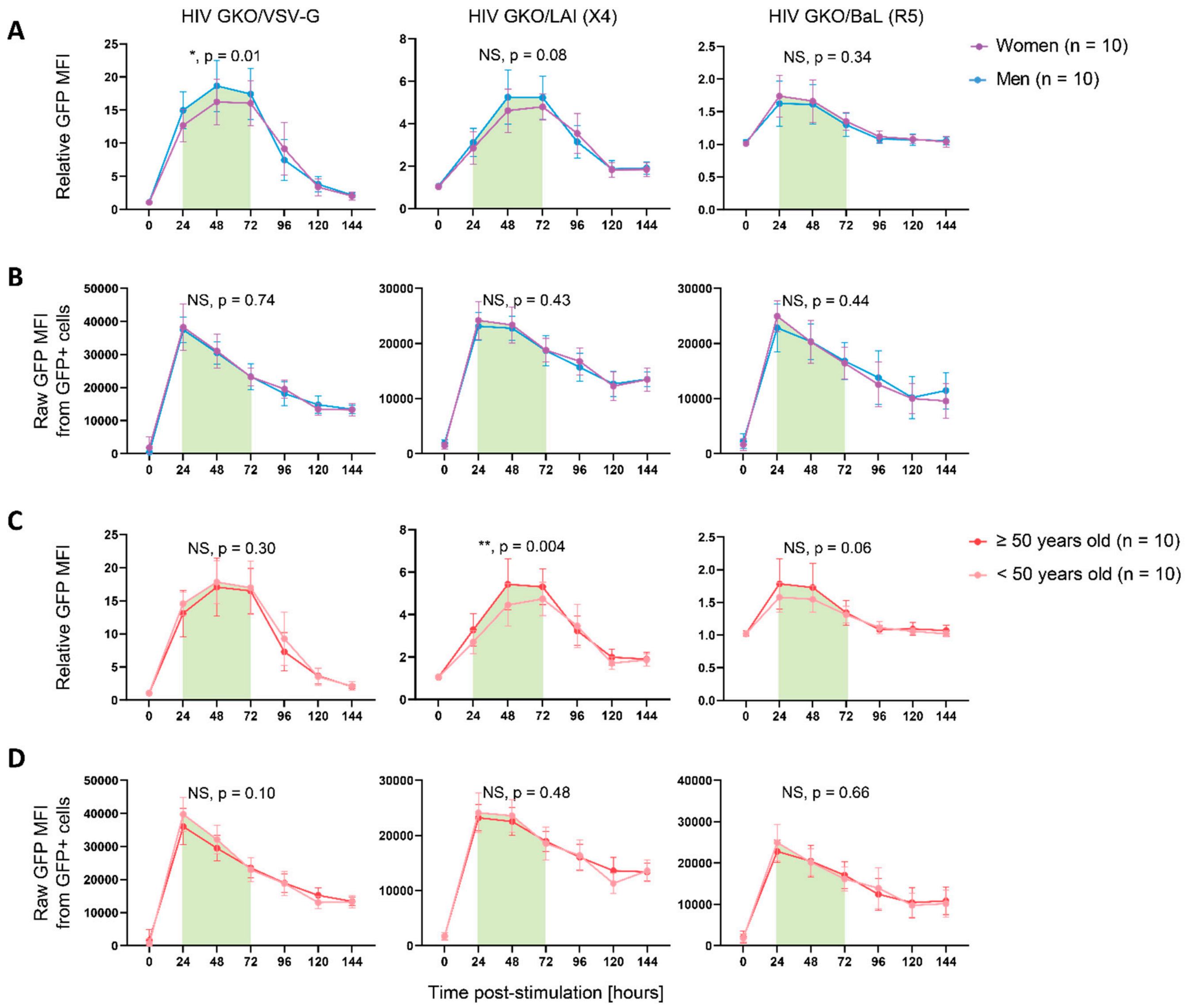

Figure S4. Sex and age impact cell permissiveness to HIV infection at the population level but not at the intracellular level. Permissiveness to HIV over time p.-s. for the three HIV GKO reporters (VSV-G, LAI (X4), BaL (R5)), separated according to sex (women: purple; men: blue) (**A, B**) or according to age (< 50 years old: pale orange; ≥ 50 years old: red) (**C, D**). Lines represent the mean of each category per time point. Error bars represent SD. Statistical differences were calculated between donor groups during the 24 and 72h p.-s. time window (indicated by the green box) using two-way ANOVA. Global cell population permissiveness to HIV was assessed as relative GFP MFI, normalized by the corresponding mock-infected control (**A, C**) and intracellular permissiveness level was assessed by measuring the raw GFP MFI in GFP+ cells (**B, D**).

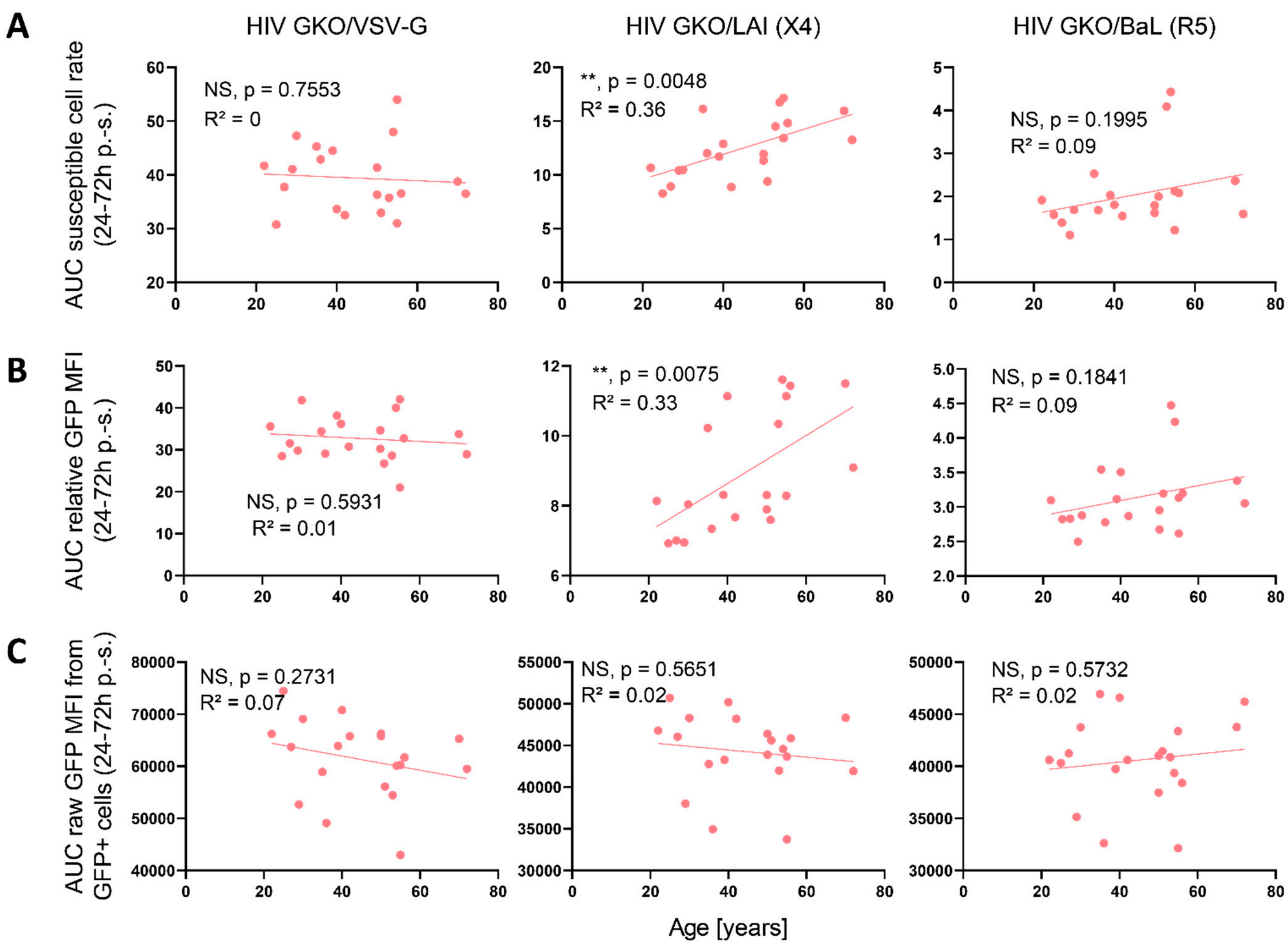

Figure S5. Age correlates with HIV infection success upon LAI (X4)-mediated entry. HIV infection success is represented by AUC 24 and 72h p.-s of susceptible cell rate (**A**), global permissiveness to HIV reflected by GFP levels (**B**) and intracellular GFP levels (**C**). Each dot corresponds to the mean of biological duplicates for one donor. Correlation with donor age was calculated for the three HIV GKO vectors (VSV-G, LAI (X4), BaL (R5)) using linear regression.

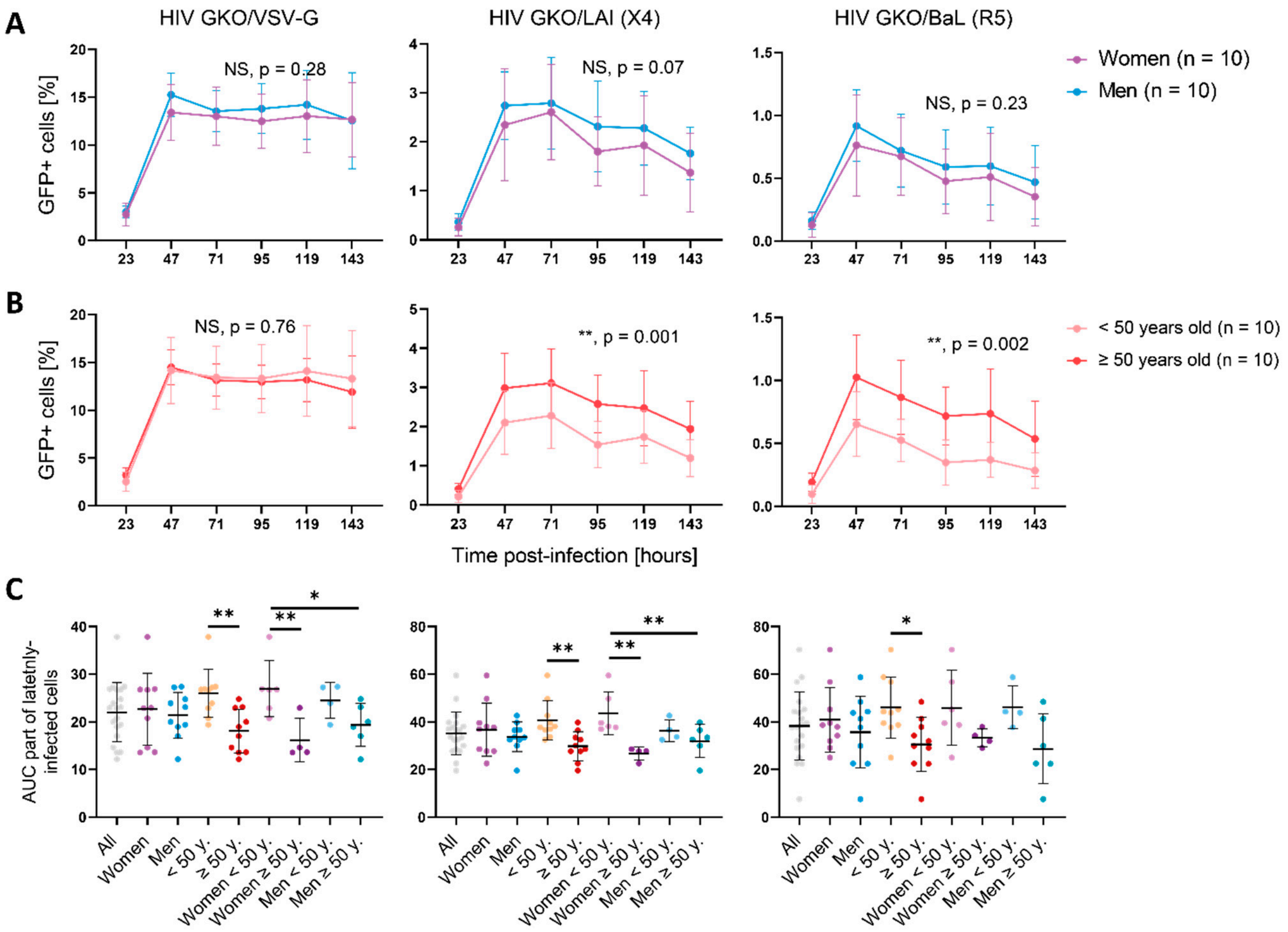

Figure S6. Sex and age impact latency establishment over time post-infection. (**A, B**) Stability of the proportion of productively infected cells (GFP+) over time p.-i. upon infection with the three HIV GKO reporters (VSV-G, LAI (X4), BaL (R5)), according to sex (**A**) and age (**B**). Lines represent the mean of each category per time point. Error bars represent SD. Statistical differences were calculated between donor groups using two-way ANOVA. (**C**) Proportion of latently infected cells (mKO2+ GFP- over mKO2+ population), calculated as the AUC over time p.-i. for each HIV GKO vector, in multiple donor categories. Each dot corresponds to the mean of biological duplicates of one donor. Statistical differences between sex and age were calculated by two-way ANOVA.

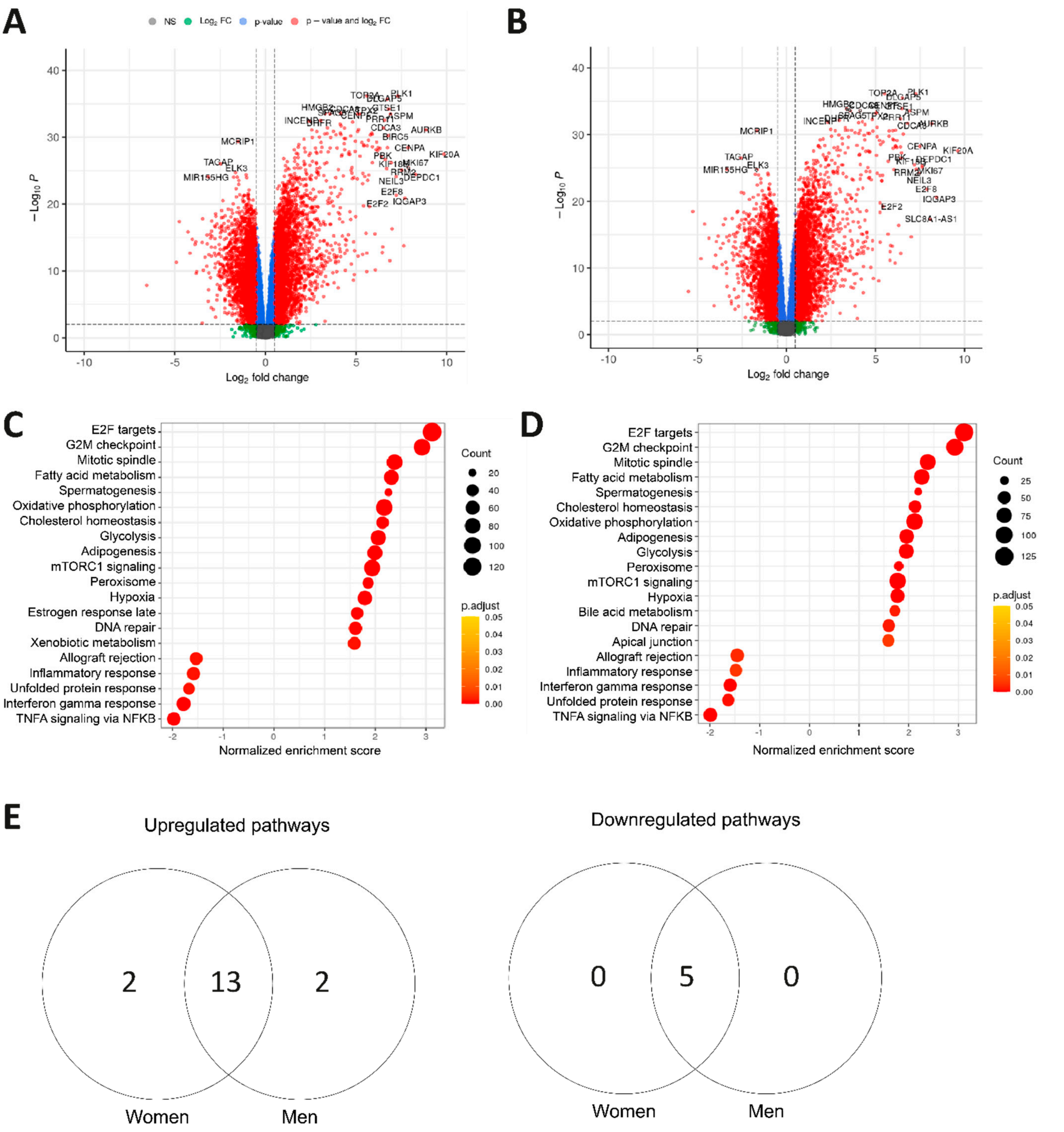

Figure S7. Transcriptome impact of 72h vs 24h stimulation. (**A, B**) Volcano plot of DEG between 72h and 24h stimulation in women (**A**) and in men (**B**) cells. Dashed lines represent cut-offs for identification of DGE ($Log_2FC > 0.5$ and $-Log_{10}$ $p > 2$). Significantly enriched DGE are represented in red, significant p-value only in blue, significant Log FC only in green and non-significant genes in grey. (**C, D**) Hallmark enrichment score between 72h and 24h stimulation in women (**C**) and in men (**D**) cells. P-values are represented by significance in orange to red shades. Gene count per pathway is represented by circle size. (**E**) Venn diagram of commonly upregulated and downregulated hallmark pathways between women and men samples.

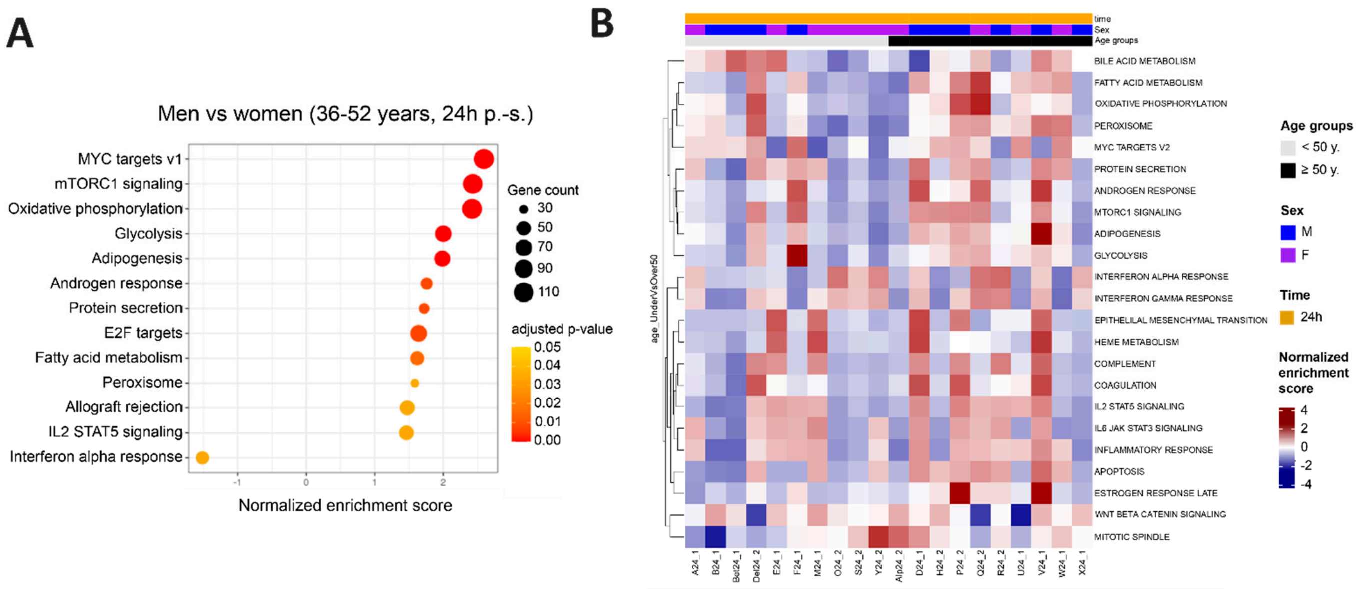

Figure S8. Impact of sex or age on functional pathways. (**A**) Hallmark enrichment score between men and women cells in 36-52 year-old age group at 24h p.-s. P-values are represented by significance in orange to red shades. Gene count per pathway is represented by circle size. (**B**) Significantly enriched hallmark gene sets between donors < 50 years and donors ≥ 50 years at 24h p.-s. Normalized enrichment score is represented in red to blue shading.

Table S1. Antibodies used for cell surface marker staining.

Target protein	Fluorophore	Host/Target Ig	Reference
CD4	APC	Rat IgG2b, κ	Biolegend, ref. 357407
CXCR4	PE	Mouse IgG2a, κ	Biolegend, ref. 306505
CCR5	PE	Rat IgG2b, κ	Biolegend, ref. 359105
CD69	PE	Mouse IgG1, κ	Biolegend, ref. 310905
CD25	APC	Mouse IgG1, κ	Biolegend, ref. 302609
HLA-DR	PE	Mouse IgG2a, κ	Biolegend, ref. 307606
PD-1	PE	Mouse IgG1, κ	Biolegend, ref. 329905
CTLA-4	APC	Mouse IgG1, κ	Biolegend, ref. 349907
TIM-3	APC	Mouse IgG1, κ	Biolegend, ref. 345011

Table S2. Comparison of statistical outcome using two-way ANOVA (as described in the main text) and paired t-test performed with paired time points, for all figures presented in the main text.

Figure	Parameter measured	Group comparison	Condition	p-value	
				Two-way ANOVA	Paired t-test
Fig. 2A	Susceptible cell fraction	Men *vs* women (24-72h p.-s.)	HIV GKO/VSV-G	****, p < 0.0001	*, p = 0.01
			HIV GKO/LAI	**, p = 0.004	*, p = 0.01
			HIV GKO/BaL	NS, p = 0.91	NS, p = 0.87
Fig. 2B	Susceptible cell fraction	< 50 y. *vs* ≥ 50 y. (24-72h p.-s.)	HIV GKO/VSV-G	NS, p = 0.70	NS, p = 0.15
			HIV GKO/LAI	***, p = 0.001	*, p = 0.01
			HIV GKO/BaL	*, p = 0.02	NS, p = 0.14
Fig. 3A	Positive cell fraction	Men *vs* women	CD4	NS, p = 0.99	NS, p = 0.99
			CXCR4	NS, p = 0.95	NS, p = 0.86
			CCR5	NS, p = 0.57	NS, p = 0.46
			CD69	***, p = 0.0007	**, p = 0.003
			CD25	NS, p = 0.23	*, p = 0.03
			HLA-DR	NS, p = 0.77	NS, p = 0.16
			PD-1	**, p = 0.009	****, p < 0.0001
			CTLA-4	**, p = 0.009	**, p = 0.003
			TIM-3	NS, p = 0.05	*, p = 0.02
Fig. 3B	Positive cell fraction	< 50 y. *vs* ≥ 50 y.	CD4	***, p = 0.0002	*, p = 0.01
			CXCR4	*, p = 0.02	*, p = 0.02
			CCR5	NS, p = 0.19	NS, p = 0.14
			CD69	NS, p = 0.90	NS, p = 0.87
			CD25	NS, p = 0.07	*, p = 0.02
			HLA-DR	NS, p = 0.17	***, p = 0.0002
			PD-1	NS, p = 0.49	NS, p = 0.11
			CTLA-4	*, p = 0.01	**, p = 0.007
			TIM-3	NS, p = 0.17	NS, p = 0.06
Fig. S4A	Relative GFP MFI	Men *vs* women (24-72h p.-s.)	HIV GKO/VSV-G	*, p = 0.01	*, p = 0.02
			HIV GKO/LAI	NS, p = 0.08	NS, p = 0.05
			HIV GKO/BaL	NS, p = 0.34	NS, p = 0.07
Fig. S4B	Raw GFP MFI from GFP+ cells	Men *vs* women (24-72h p.-s.)	HIV GKO/VSV-G	NS, p = 0.74	NS, p = 0.26
			HIV GKO/LAI	NS, p = 0.43	NS, p = 0.17
			HIV GKO/BaL	NS, p = 0.44	NS, p = 0.55
Fig. S4C	Relative GFP MFI	< 50 y. *vs* ≥ 50 y. (24-72h p.-s.)	HIV GKO/VSV-G	NS, p = 0.30	NS, p = 0.09
			HIV GKO/LAI	**, p = 0.004	*, p = 0.03
			HIV GKO/BaL	NS, p = 0.06	NS, p = 0.12
Fig. S4D	Raw GFP MFI from GFP+ cells	< 50 y. *vs* ≥ 50 y. (24-72h p.-s.)	HIV GKO/VSV-G	NS, p = 0.10	NS, p = 0.27
			HIV GKO/LAI	NS, p = 0.48	NS, p = 0.37
			HIV GKO/BaL	NS, p = 0.66	NS, p = 0.76
Fig. S6A	GFP+ cell fraction	Men *vs* women	HIV GKO/VSV-G	NS, p = 0.28	*, p = 0.03
			HIV GKO/LAI	NS, p = 0.07	**, p = 0.003
			HIV GKO/BaL	NS, p = 0.23	**, p = 0.004
Fig. S6B	GFP+ cell fraction	< 50 y. *vs* ≥ 50 y.	HIV GKO/VSV-G	NS, p = 0.76	NS, p = 0.34
			HIV GKO/LAI	**, p = 0.001	**, p = 0.001
			HIV GKO/BaL	**, p = 0.002	**, p = 0.001

Table S3. Cell subtype distribution in four selected donors, based on three annotated reference datasets for comparison. Raw cell counts and frequency distribution (percentage) over total population are indicated for each comparison. Sex, age and total cell count are indicated for each donor.

**Reference**	**Subset**	**Donor 1 (M, 29 y., 6601 cells)**		**Donor 2 (M, 36 y., 5802 cells)**		**Donor 3 (F, 25 y., 6288 cells)**		**Donor 4 (F, 39 y., 5878 cells)**	
		**Count**	**Freq.**	**Count**	**Freq.**	**Count**	**Freq.**	**Count**	**Freq.**
**Seurat [26]**	CTL	85	1.3	281	4.8	89	1.4	188	3.2
	CD4 Naive	337	5.1	500	8.6	460	7.3	646	11.0
	CD4 Proliferating	696	10.5	429	7.4	305	4.9	446	7.6
	CD4 TCM	4992	75.6	4084	70.4	4636	73.7	4198	71.4
	CD4 TEM	34	0.5	90	1.6	263	4.2	56	1.0
	Treg	457	6.9	418	7.2	535	8.5	344	5.9
**Monaco [27]**	Follicular helper	243	3.7	413	7.1	268	4.3	415	7.1
	Naïve CD4	852	12.9	935	16.1	732	11.6	1133	19.3
	T regulatory	2044	31.0	1282	22.1	1595	25.4	1204	20.5
	Terminal effector CD4	141	2.1	297	5.1	121	1.9	232	3.9
	Th1	1193	18.1	725	12.5	808	12.8	884	15.0
	Th1/Th17	1647	25.0	1623	28.0	2171	34.5	1477	25.1
	Th17	233	3.5	289	5.0	293	4.7	227	3.9
	Th2	248	3.8	238	4.1	300	4.8	306	5.2
**DICE [28]**	Memory Treg	447	6.8	436	7.5	546	8.7	409	7.0
	Naïve	239	3.6	380	6.5	142	2.3	465	7.9
	Naïve Treg	56	0.8	155	2.7	84	1.3	105	1.8
	Naïve stimulated	4946	74.9	3488	60.1	4410	70.1	3537	60.2
	Tfh	501	7.6	766	13.2	524	8.3	761	12.9
	Th1	164	2.5	272	4.7	242	3.8	317	5.4
	Th1/Th17	57	0.9	89	1.5	138	2.2	51	0.9
	Th17	111	1.7	136	2.3	131	2.1	99	1.7
	Th2	80	1.2	80	1.4	71	1.1	134	2.3
